# Psychological process from hospitalization to death among uninformed terminal liver cancer patients in Japan

**DOI:** 10.1186/1472-684X-5-6

**Published:** 2006-09-04

**Authors:** Yuko Maeda, Akihito Hagihara, Eiko Kobori, Takeo Nakayama

**Affiliations:** 1 Kyoto University School of Public Health, Kyoto, Japan; 2 Graduate School of Medical Science, Kyushu University, Fukuoka, Japan

## Abstract

**Background:**

Although the attitude among doctors toward disclosing a cancer diagnosis is becoming more positive, informing patients of their disease has not yet become a common practice in Japan. We examined the psychological process, from hospitalization until death, among uninformed terminal cancer patients in Japan, and developed a psychological model.

**Methods:**

Terminal cancer patients hospitalized during the recruiting period voluntarily participated in in-depth interviews. The data were analyzed by grounded theory.

**Results:**

Of the 87 uninformed participants at the time of hospitalization, 67% (N = 59) died without being informed of their diagnosis. All were male, 51–66 years of age, and all experienced five psychological stages: anxiety and puzzlement, suspicion and denial, certainty, preparation, and acceptance. At the end of each stage, obvious and severe feelings were observed, which were called "gates." During the final acceptance stage, patients spent a peaceful time with family, even talking about their dreams with family members.

**Conclusion:**

Unlike in other studies, the uninformed patients in this study accepted death peacefully, with no exceptional cases. Despite several limitations, this study showed that almost 70% of the uninformed terminal cancer patients at hospitalization died without being informed, suggesting an urgent need for culturally specific and effective terminal care services for cancer patients in Japan.

## Background

Reflecting the popularity of informing a patient of a cancer diagnosis in Western countries, a positive attitude toward the disclosure of a cancer diagnosis has been increasing in recent years among doctors and the public in Japan. Several surveys in Japan have indicated that most people, one of which showed more than 80% of all patients [1], would like to be told the true diagnosis if they were to have cancer [1-6]. Some medical doctors have accepted and gradually put the custom of informing terminally ill patients of their disease into practice, but they represent a minority [7].

Most doctors in Japan still prefer to not disclose the diagnosis to cancer patients [8]. A nationwide study on death with dignity conducted by the Health, Labor, and Welfare Ministry of Japan revealed that only 46% of terminal cancer patients had been informed of their disease, demonstrating that informing patients has not yet become a common practice in Japan [9].

Doctors report several reasons for not disclosing a cancer diagnosis to a patient. Some doctors are concerned that their training in the psychological care of informed patients may be insufficient [1,10], causing anxiety for some doctors that they may be unable to manage the potential depression in terminal patients upon disclosure and that their relationship with the patient might be adversely affected [1]. Families as well as doctors display an aversion to taking responsibility for the potential risks of disclosure [11]. The Japanese norm based on the traditional obligations of family and health professionals has resulted in the practice of not disclosing information or a diagnosis to the patient [12,13].

In the United States, the psychological processes through which informed patients pass were examined in a study by Kubler-Ross [14]. A study by Glaser and Strauss identified four types of content awareness-*Closed awareness, Suspicion awareness, Mutual pretence and Open awareness*-based on the relationship between patients and the people around them [15]. In Japan, the psychological processes experienced by uninformed patients were described by Kashiwagi and were based on open discussions among doctors and nurses on death and dying in 1990s [16]. This study identified the first four psychological processes of uninformed terminal cancer patients as *hope*, *suspicion*, *anxiety*, and *depression*; in the fifth stage, the patients were categorized into two groups: *gave up *or *accepted death*. These findings contribute to an understanding of the psychological processes experienced by terminal cancer patients; however, a more verifiable study is necessary to establish culturally specific and effective psychological care for terminally ill patients in Japan.

The purpose of this study was to examine the psychological processes occurring from hospitalization to death among uninformed middle-aged male terminal liver cancer patients in Japan. Understanding the psychological processes of uninformed patients could provide critical insight for developing relevant psychological care for patients and families with cancer.

## Methods

### Participants

We recruited terminal liver cancer patients who were hospitalized in the Department of Surgery at a hospital in Hyogo Prefecture from December 1998 to June 2002. In accordance with hospital policy, none of the patients had been informed of their diagnosis or prognosis. Consequently, the purpose of this study could not be explained completely to the patients. This will be discussed in detail in the data collection section below. This study, including how patients should be informed of its purpose, was approved by the ethics committees of the hospital, the study site, and Osaka Women's University.

### Data collection

All of the interviews were conducted by the first author (Maeda) throughout the study. The interviewer introduced herself to the patients hospitalized during the recruitment period and explained that she was interested in studying the psychological process of hospitalized patients. It was further explained that the patients were welcome to come and talk with the interviewer, who would be standing visibly at the nurses' station, at any time and about whatever they would like.

The interviewer waited for patients to voluntarily approach her, respecting their voluntary decision to participate. When the patients came to talk, they were told about the study based on the informed consent form, which described the study as a patient satisfaction study focused on psychological aspects. Patients were further informed that the conversation would be tape-recorded during the interview but that the confidentiality and anonymity of the participants would be securely maintained. After obtaining a signed consent form, an in-depth interview was conducted in an isolated room.

With regard to not fully explaining the study purpose to patients because they were unaware of their diagnosis, we finally concluded that, although it might present ethical concerns, it seemed to be the only possible approach and to be justifiable given that the findings from this study could be beneficial in providing more appropriate psychological support to terminally ill patients.

To deal with this dilemma during the interview, the interviewer listened to the participants talk on their own initiative and was careful to express her response in nonverbal ways, such as nodding to show interest or tilting her head a little to one side to indicate confusion. This method seemed the most suitable approach to avoid disturbing the patient's psychological flow, keeping the flow as natural as possible. The primary interest of the study, to identify psychological processes, was addressed through at least 15 one-on-one interviews with each patient; each interview session was about 1.5 hours in duration.

### Analysis

The analysis was conducted immediately following the interview by adopting a grounded theory from Cobin and Strauss [17], which has the ability to identify social interaction processes using a data-oriented analysis [18-20]. The data analysis started with open, axial and selective coding. Open coding included repeated readings of the interviews and in-depth, line-by-line analysis of the data. Using of open coding, data were coded under various headings according to their content with the purpose of opening up data as well as achieving a constant comparison of incidents and categories that emerged from subsequent interview. By means of these codes, we developed several categories and subcategories. The axial coding linked categories and subcategories together. In the final selective coding, the core category was identified from among the linked categories; and the core category and the other categories were sorted out and linked, which resulted in developing concepts that consisted of the psychological process.

Multidisciplinary members participated in the analysis and discussions conducted through regular meetings among the first author, whose background was social psychology and who also served as the interviewer, and other the members, who were specialized in qualitative research methodology. After the data collection was completed, all of the data were then discussed and reviewed by additional team members specialized in public health and medicine and the first author. To contribute to the validity of the study result, the analysis was conducted by multidisciplinary members including the interviewer and other members of whom did not, participated in the interviews.

The interviews were transcribed verbatim by a professional transcriptionist. After all of the data were completed, typical and appropriate examples of conversations were selected for each process and translated into English, using words and expressions carefully selected by the social psychologist. After the translated conversations were checked by a native speaker of English, the appropriateness of the translation in substantially conveying the original sensitive psychological information was further examined. After several corrections from this perspective, the English conversations were again checked by native English speakers.

## Results

Of the 87 terminal liver cancer patients hospitalized during the recruiting period without being informed of their diagnosis or prognosis, 67% (N = 59) died without knowing their diagnosis. The remaining 28 patients were excluded from the analysis; 25 patients learned of their diagnosis by asking either medical staff or family members before they died, and three patients died in earlier stages without being suspicious about their disease. Table 1 shows the characteristics of the 59 participants who died without disclosure. The patients, all male and ranging in age from 51 to 66 years, were highly uniform in terms of gender, age, profession, and education.

**Table 1:** Participant characteristics (N = 59)

Male/Female	59/0
Age (years) (mean ± SD)	58.7 ± 3.53
Type of living	
Living with wife	55
Living without wife or alone	4
Religious belief	
Don't have any special beliefs	52
Have beliefs	7
Education	
University graduate or higher	52
High school or Vocational school graduate	7
Occupation	
Office worker	33
Company executive	8
Retired	7
Farmer	6
Specialist	5
Period of hospitalization (month)	
Mean ± SD	19.2 ± 1.5
Range	14.2–21.6
Period from hospitalization to the first interview (day)	
Medican (25 percentile-75 percentile)	8 (6–12)
Period from the last interview to death (day)	
Medican (25 percentile-75 percentile)	8(5–11)

In the study, a large number of participants were interviewed given that saturation occurred with much fewer participants. The reason why we recruited and interviewed many more participants than the number necessary for the saturation was related to the issue of time lag between the time of patient's recruitment and of the completion of an interview. Specifically, regarding saturation, it was not until the last moment (death) that patient's entire psychological process and if he was informed of his diagnosis or died without being informed could be identified. In reality, 18 months had already passed from his recruitment until death; during which a number of patients had been recruited and interviewed. In addition, the hospital asked us to continue the interview until the last moment once a patient was recruited and started the interview process. In terms of the ethical implications, however, once saturation was confirmed, we should have stopped the additional interviews and data analysis based upon those recruits.

From the time of hospitalization until death, five psychological stages, consisting of 24 processes, were identified during the entire psychological process of the participants. All 24 processes and typical excerpts for each are presented in Table 2. Unlike the other processes, four of them, although short-term, were characterized by the obvious and severe expression of feelings and behaviors, as shown in bold in Table 2. All 57 participants passed through all 24 processes with no backtracking or omissions.

**Table 2:** Dialogues in each process

**Stage 1**
*Process 1. Restriction in daily life*
"It's disturbing that I'm not allowed to smoke..." (55) "I'll become even more sick if I have to live without alcohol!" (56) "It isn't surprising that I can't fall asleep easily because I am forced to sleep earlier than usual every day, don't you think?" (59) "Oh, God! I can't stand this life! You can't understand it!" (63)
*Process 2. Financial burden*
"I was hospitalized just for a medical checkup, so I'll be out of here soon. Even so, it will cost me a lot with all the medical equipment they're using to check me over... Why was I put into the hospital? I don't feel sick anywhere in my body... I know my body best. Doctors try to rob patients of money by hospitalizing us regardless of the real condition of our health!" (57)
*Process 3. Restriction in physical conditions (Sensing deteriorated health)*
"I can't eat a full meal." (63) "I want to lie down on the bed." (54) "I have a fever." (56) "I become fatigued and feel like shouting." (58)
*Process 4. Problems in health communication*
"Something is wrong. I can't quite understand what my doctor says. He told me something difficult to understand. His explanation for my symptom is not clear to me." (61) "His explanation was not clear!" (61)
*Process 5. Anxiety about being abandoned*
"These days, my family doesn't visit me as often as before. Although they come to see me, they don't talk much. I feel uneasy. I feel they are avoiding me. I wish they were here when I'm in pain... You are the only one who listens to me all the time." (59)
*Process 6. Anxiety about being a sick*
"I've just launched an important project. I have to take responsibility if it doesn't work out properly. For now, I can communicate with those involved via e-mail, but once they know I am sick, it will cause a big commotion. After a while, I want to go back to my company to let them know I am OK." (57) "I told my children I am in the hospital for a medical checkup. My eldest child is preparing for an entrance exam. I can't afford to be sick anyway. I have to go home as soon as possible." (54)
*Process 7. Anxiety about being hospitalized*
"Every day, my wife comes to the hospital to take care of me. I have a daughter living at home, but she is not available because she has to go to school. So, I am worried about my wife's health, since she has to take care of both the household chores and me." (61)
***Process 8. Anger (Not being advised of the condition of their health***
"Something is wrong with my body. Even so, my family doesn't tell me anything about it. Am I seriously ill? Doctors and nurses don't give satisfactory answers to my questions, either." (55)
Period of the stage counted from hospitalization: 0.2–1.7 months
**Stage 2**
*Process 9. Death perspectives thinking about the unknown (Talking about death)*
"Patients tend to think about what death would be like." (59) "Death is not predictable." (63) "We know the death is next to us all the time." (58)
*Process 10. Shame*
"If I am found to suffer from such a disease, it would affect my daughter's marriage, and it would be shameful for my family to be thought of as physically weak." (52)
*Process 11. Death Perspectives – Frustration/Separation*
"If I die now, my family will have a difficult life." (58) "It's unbearable if I can't do well enough for my family." (59) "If I died now, I wonder what my life would have been. The plan for my life would crumble. My dreams and hopes would be dashed!" (60)
***Process 12. Refusal (Rejection of family and medical staff)***
"My family and doctors alike seem to be hiding something from me. My physical condition isn't good, despite what the results of the medical checkup indicate. Ms. Maeda, do you know something about me? Did you hear something? I wonder if I have to go through surgery? If so, I want it earlier than later. I'm not going to ask my family and doctors because they only repeat the same answers... Ms. Maeda, if you have some information about me, please let me know in private. I won't question anyone else about it anymore, just you." (61)
Period of the stage counted from hospitalization: 1.0–6.0 months

**Stage 3**
*Process 13. Loneliness-Anticipating death*
"Death does not matter to me." (62) How meaningful is it for me to live longer in such ill health?" (54) "To die is very sad." (56) "When you die, you are alone. Death is the last unfortunate event in life." (56) "We are alone, alone, alone........when the death is coming to us." (57)
*Process 14. Anxiety about medical test and treatments*
"They just say 'it's a checkup, it's a checkup' and get me to go through more check-ups, which are painful in many ways. I have never experienced checkups like this before and they are really unbearable. If this situation continues, I feel like my body will break down with all these check-ups and treatment!" (62)
*Process 15. Fear of death*
"I feel uneasy when I think of my death." (60) "I am suffering from cancer. I am scared and worried about dying in agony." (61)
*Process 16. Emptiness*
"If I die, will I be forgotten?" (57) "My death would be meaningless for society as a whole." (63) "Even if I died, the world would not change at all..." (55)
***Process 17. Perceived approaching death***
"You remember, Mr. XX, who was hospitalized in the same room? That guy passed away last night. When I told my wife, I said, 'His family had difficulties when he was alive. From now on, his wife will lead an easier life.' My wife had a slightly angry expression on her face when she replied, 'How can you tell she would lead an easier life?'." (63) "Humans have a really ephemeral existence. Before, I felt I would die whenever somebody around me passed away. However, now I feel it really close to me. Having said that, in reality, it isn't possible to feel our own death until the last minute, as long as we are in this life..." (61)
Period of the stage counted from hospitalization: 4.0–9.7 months

**Stage 4**
*Process 18. Resigned to death*
"Knowing how to die reflects a person's attitude/thinking towards life." (55) "Only when I die will the value of my life be clear." (58) "Death is the important and decisive moment for people." (62) "I think people become perfect or complete at the moment they pass away." (60)
*Process 19. Thinking about the next life*
"After I die, I will return to this world by reincarnation." (62) "When people die, they will be reborn in another world. I just wonder whether I could have a good life then." (63) "I wonder if I could go to a place where I would be more satisfied when I die." (54)
***Process 20. Grief***
"........."
Period of the stage counted from hospitalization: 7.5–15.1 months

**Stage 5**
*Process 21. Reconfirmation of their own lives*
"My family depends on me." (66) "My friends depend on me." (54) "I feel happy now." (58) "I feel that I have been helping others so far." (59) "I have tackled everything very aggressively up until now." (60) "I have to be a reliable person. After my hospitalization, my families are united." (54)
*Process 22. Good memories*
"There were some events in my life that are too painful to remember." (55) "When I look back on my life, I feel very satisfied." (65) "When I remember the past, I'm encouraged and feel peaceful." (57) "When I look back on my life, my heart is full." (61)
*Process 23. Appreciation*
"I am really grateful to my wife for everything. I am worried that my wife may fall ill because of me..." (57) "I appreciate everybody for not telling me that I have cancer up until the last minute..." (62) "Ms. Maeda, thank you for having heard my complaints. Without you, my hospitalization might have been much different. You might have had a very difficult time. Did you get learn something by interviewing me?" (59)
*Process 24. Wishes*
"If I could be out of the hospital, let's do this and that, shall we?" (56) "I would like to see the first sunrise for the year on New Year's Day next year on the top of Mt. Fuji." (59) "Let's go on a family trip!" (61)
Period of the stage counted from hospitalization: 12.6–21.1 months

Numbers in the parenthesis are the ages of the person.

### Stage 1

This stage began immediately after hospitalization and continued through the second or third week as an inpatient. The patients felt anxious about the limitations, such as financial burdens, restrictions in daily life, and physical conditions resulting from their hospitalization, which had suddenly and totally changed the daily lives. They were puzzled by the gap between the explanations offered by the doctor and the family and the reality they were feeling. At the end of this stage, patients reached the point of expressing their feelings, and anger showed clearly and strongly in their faces and behaviors. The patients expressed anxiety related to the way in which their family and the medical staff did not provide clear information regarding their diagnosis.

### Stage 2

In this stage, the patients began to talk about death, but only in general. They were suspicious that their disease was cancer and felt ashamed because cancer has been regarded as special and looked upon negatively in the Japanese culture. Then, they assumed they were dying and expressed their frustration and anxiety with their life and family members. At the end of this stage, patients expressed their feelings clearly and strongly. They exhibited irritation regarding the conflict between their perception of their deteriorating physical condition and the unclear information they had been given. The patients flatly refused to communicate with families and medical staff and instead attached hope to the interviewer as a third party who might know something.

### Stage 3

At this stage, the patients voiced feelings of loneliness and anxiety about the pain caused by various unfamiliar medical tests. However, after about the seventh month of hospitalization, the patients avoided talking about death in general. They clearly expressed their recognition that they were suffering from cancer and conveyed to the interviewer their fear that they might no longer be able to escape death, which resulted in feelings of emptiness. At the end of this stage, the patients frequently looked for the opportunity to talk with their families, and once-disrupted communication returned to normal. The patients started to talk about the deaths of significant others and perceived their own time drawing near. They further presented their own views of death and gleaned information about their disease and the possibility of death from the reactions and attitudes of their families and the people around them.

### Stage 4

In this stage, the patients tried to find some implication of their death, which moved them to the idea of the next world or life after death. At the end of this stage, the patients were overwhelmed by grief, with tears and silence filling many minutes or with almost an hour spent just sitting in front of the interviewer. It was as if pent-up steam was being vented all at once after making efforts to find as much affirmative meaning as possible in death. The patients realized that they could not escaped from death.

### Stage 5

The patients went through a process of confirming their reason for being in relation to the people close to them and took the opportunity to look back upon their own life again by talking with family members about various memories. The patients started voicing words or statements that could be taken as farewells and gratitude to the people around them. They also cared about the health of their family. Finally, the patients talked about their wishes in a warmhearted atmosphere with their family. In this stage, the patients made no references to death.

#### Model development of the psychological process

**Figure 1:**
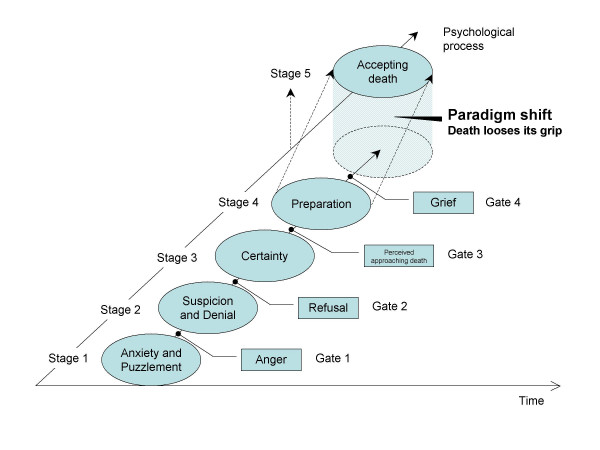
Psychological process model.

Based on the result, we modeled the psychological process among uninformed male cancer patients, from hospitalization until death. (Figure 1)

Of the 24 processes, we first focused on four that involved clear and severe feelings, indicating peaks of feelings and behaviors caused by the feelings accumulated during the previous processes. These four processes facilitated the transition to the next phase, and thus we called these four processes 'gates,' to signify the passage to a different level. We then categorized all 24 processes into five 'stages' by placing the gate as a boundary between each stage. The gate is therefore defined as the endpoint of a psychological stage in which a patient's feelings have accumulated and have been released, as well as the node point leading to the next psychological stage.

##### Stage 1: anxiety and puzzlement

In the 'anxiety and puzzlement' stage, patients are anxious about various issues because of the unseen future resulting from sudden hospitalization. Patients are still unaware of their serious health condition and are thus puzzled by the number of medical tests being performed, many of which are normally associated with seriously ill patients. The anxiety and puzzlement can never be resolved at this stage because patients have not been informed of the results of the diagnosis. This situation gradually causes patients to exceed the threshold of their patience and moves patients to gate 1, named 'anger,' when the patients' anxiety is expressed as anger toward family members and medical staff.

##### Stage 2: suspicion and denial

In this stage, patients suspect with some irritation that they might be dying or might have a serious disease. They have unfulfilled feelings toward what they are facing and begin talking about their perspectives on death. Their feelings cause emotional conflict such as frustration or irritation because they are not given adequate information from family and medical staff. At the end of this stage, patients deny the comfort of family and refuse to communicate with people around them, passing through the gate named 'refusal.'

##### Stage 3: certainty

As a consequence of suspicion and denial, the patients' psychological condition moves into the stage named 'certainty,' which refers to the certainty of death. Without being informed of their diagnosis, patients are left alone with suspicions of being seriously ill and with mercilessly continued medical examinations and treatments, which convince them of their suspicions. Patients are seized with a fear of death and the emptiness of life. As a result of these feelings, however, the once-disrupted communication with family and others around them is resolved, and patients frequently mention the death of significant others. This last process, named 'perceived approaching death,' is gate 3.

##### Stage 4: preparation

As they prepare for resigning to death from cancer, the patients talk about their own life and death. They make an effort to view death positively, struggle against fear, and begin preparing for their own death. Then, patients release built-up emotions all at once with a heavy silence and tears, as fear produces uncontrollable grief, gate 4, about accepting their own death.

##### Stage 5: acceptance

After patients pass through gate 4, the process of deep grief, the paradigm of death and life changes dramatically for patients. Death seems to loose its grip on the mind, and it is not spoken of anymore, although thoughts of death never disappear. Patients look back over their career and life at what they can be proud of and become close to their family, who has always been with them. They have a strong desire for warmhearted times and even talk about their dreams with their family, although they might know that the dreams will never be realized. In this stage, patients spend time peacefully appreciating the people around them, gradually deepening their level of acceptance.

## Discussion

In this study, almost 70% of the Japanese male terminal liver cancer patients who had not been informed of their diagnosis and prognosis at the time of hospitalization were never informed before death. We identified the psychological process of those patients, from the time of hospitalization until death, during which the patients passed through five stages and four gates to arrive at a peaceful acceptance of death. Based on the results, we developed a model of the psychological process.

Kubler-Ross developed a psychological process model for terminal cancer patients in the United States, and Kashiwagi described a similar process in Japanese patients [14,16,21]. Both collected their data through their daily experiences as medical doctors providing care and treatment for terminally ill patients. Comparing our findings with those of Kubler-Ross may be problematic because the disclosure status was different between the two target patient groups; nevertheless, a major difference in the patient's psychological condition appears to occur during the last stage. The informed patients of Kubler-Ross were not happy and were almost void of feelings at the "acceptance" stage, whereas the uninformed patients in this study had peaceful feelings about accepting death. Kashiwagi identified the patients were categorized into two groups, those who gave up in a negative sense and those who accepted death in a positive sense in the last stage. By contrast, all of the patients in the present study arrived at the same last stage of peaceful acceptance, which might be explained by the high degree of similarity among the patients in this study in terms of sex, age, and education, as well as disease. Kashiwagi is assumed to have identified psychological processes by observing various types of patients.

In addition to the effect that the similarities among the patients in this study had on the finding of a positive attitude toward a peaceful death in all patients, the traditions and customs of the Japanese culture, which values the relationships and responsibilities of family, would have fostered a positive attitude toward death. Japanese society is a collectivistic or interdependent culture, which establishes and maintains interdependence among individuals [22,23]. Individuals in such cultures are socialized to adjust to an attendant relationship or a group to which they belong, to read one another's minds, to be sympathetic, to occupy and fulfill their assigned roles, and to engage in appropriate actions [24-27]. This mind-set is reflected in the finding that when patients perceived approaching death at the third gate, they talked about the deaths of significant others. The families fulfilled their culturally assigned role, listening for the thoughts and desires that the patients were trying to express. Most of the patients in this study, by virtue of being middle-aged men in Japan, supported their families financially and emotionally, and family relationships had priority over any other associations for these patients. Therefore, the patients strived to, above all else, value the thoughts of the family, that nondisclosure of the diagnosis was the best choice for the patient and the family in working to accept death.

One distinctive feature of the study is the potential influence of the interviewer on the patients' psychological condition. This influence became obvious when a patient expressed his appreciation to the interviewer during the last stage of his life. Given that the interviewer was a neutral party and not family or medical personnel, talking with the interviewer might have provided an opportunity for the patients to understand and reorganize themselves in their new situation, which may have facilitated the patients' psychological progression. The data collection phase had to be carefully conducted, keeping in mind that the patients were uninformed about their diagnosis. The interviews were completely dependent upon the patients' feelings and convenience. The interviewer was always careful to avoid expressing any information or indication, either consciously or unconsciously, regarding the health condition of the patients. When patients asked about their condition during an interview, they were told by the interviewer that such information would not be disclosed to a third party. In fact, no individual's medical information was disclosed to the interviewer, although the interviewer understood that all of the patients hospitalized in the department of the study site were in the terminal stage of their disease. Being in this situation caused great emotional conflict and stress for the interviewer.

This study has several limitations, related primarily to the data collection method. The observation that none of the study patients moved back and forth between stages or skipped a stage seems quite unusual because we empirically know that psychological conditions do not change unidirectionally. We therefore concluded that the interview process itself might have provided an opportunity for patients to understand and reorganize their thoughts and feelings, pushing them forward psychologically rather than leaving them to work through their feelings without direction. In addition, the high uniformity in age, sex, and education among the patients may have contributed to the uniformity of their psychological progression. Unlike the two previous studies [14,16], our study could not verify a process of depression, although we perceived its potential at the grief process in which patients were silent for long time periods. Depression may not have detected in this study because the interviewer, being a third party, did not have access to the patients' medical information, which might have shown evidence of depression. In addition, because the timing of the interviews was determined solely by the patients, it may be that patients did not come forward to talk when they were noticeably depressed. Although the high uniformity of the patients might have contributed to identifying population-specific psychological processes, it also restricts the generalizability of these study results.

## Conclusion

This study identified the psychological process, from the time of hospitalization until death, among uninformed male terminal cancer patients in Japan, which had not been well described previously. Given that half of the terminal cancer patients in Japan will remain uninformed until death and that more time seems to be required for patient disclosure to be widely accepted in Japanese culture owing to its unique family-individual relationships, exploring appropriate terminal care services for uninformed patients is an urgent need in Japan. This urgency is demonstrated by the fact that almost 70% of the patients in this study who were uninformed at hospitalization remained uninformed until death. The finding that those patients spent a peaceful last stage before death suggests that having opportunities to talk with a third party may have positive implications for those patients in a hospital setting, which may be an option for improving social palliative care service in Japan.

## Competing interests

The author(s) declare they have no competing interests.

## Authors' contributions

YM coordinated the study, participated in research design, conducted the interview, and confirmatory qualitative analysis and the manuscript writing. AH and EK and TN participated in the research design, the primary and confirmatory qualitative analysis and conceived the study and commented on the manuscript. All authors read and approved the final manuscript.

## Pre-publication history

The pre-publication history for this paper can be accessed here:



## References

[B1] Seo M, Tamura K, Shijo H, Morioka E, Ikegame C, Hirasako K (2000). Telling the diagnosis to cancer patients in Japan: attitude and perception of patients, physicians and nurses. Palliat Med.

[B2] Ruhnke GW, Wilson SR, Akamatsu T, Kinoue T, Takashima Y, Goldstein MK, Koenig BA, Hornberger JC, Raffin TA (2000). Ethical decision making and patient autonomy: a comparison of physicians and patients in Japan and the United States. Chest.

[B3] Hamajima N, Tajima K, Morishita M, Hyodo C, Sakakibara N, Kawai C, Moritaka S (1996). Patients' expectations of information provided at cancer hospitals in Japan. Jpn J Clin Oncol.

[B4] Kawakami S, Arai G, Ueda K, Murai Y, Yokomichi H, Aoshima M, Takagi K (2001). Physician's attitudes towards disclosure of cancer diagnosis to elderly patients: a report from Tokyo, Japan. Arch Gerontol Geriatr.

[B5] Uchitomi Y, Okamura H, Minagawa H, Kugaya A, Fukue M, Kagaya A, Oomori N, Yamawaki S (1995). A survey of Japanese physicians' attitudes and practice in caring for terminally ill cancer patients. Psychiatry Clin Neurosci.

[B6] Uchitomi Y (1999). Psycho-oncology in Japan: history, current problems and future aspect. Jpn J Clin Oncol.

[B7] Tanida N (1994). Japanese attitudes towards truth disclosure in cancer. Scand J Soc Med.

[B8] Horikawa N, Yamazaki T, Sagawa M, Nagata T (1999). The disclosure of information to cancer patients and its relationship to their mental state in a consultation-liaison psychiatry setting in Japan. Gen Hosp Psychiatry.

[B9] Ministry of Health, Labour and Welfare, Japan (2005). Death with dignity questionaire research.

[B10] Elwyn TS, Fetters MD, Gorenflo W, Tsuda T (1998). Cancer disclosure in Japan: historical comparisons, current practices. Soc Sci Med.

[B11] Elwyn TS, Fetters MD, Sasaki H, Tsuda T (2002). Responsibility and cancer disclosure in Japan. Soc Sci Med.

[B12] Gotay CC, Shimizu H, Muraoka M, Ishihara Y, Tsuboi K, Ogawa H (2004). Cancer-related attitudes: A comparative study in Japan and the US. Psychooncology.

[B13] Mitchell JL (1998). Cross-cultural issues in the disclosure of cancer. Cancer Pract.

[B14] Kubler-Ross E (1969). On Death and Dying.

[B15] Glaser  B, Strauss  A (1965). Awareness of Dying.

[B16] Kashiwagi T (1999). Truth telling and palliative medicine. Intern Med.

[B17] Glaser B, Strauss A (1967). The Discovery of Grounded Theory; Strategies for Qualitatiave Research.

[B18] Silverman D (2000). Doing Qualitative Reserach: A practical Handbook.

[B19] Pope C, Mays N (1999). Qualitative Research in Health Care Second Edition.

[B20] Rice PL, Douglas E (1999). Qualitative Research Methods:A Health Focus.

[B21] Kashiwagi T (2002). Shi wo mitoru Igaku.

[B22] Doi K (1982). The Anatomy of Dependence.

[B23] Matsumoto D (2000). Culture and Psychology: People around the World (second Edition).

[B24] Davis MH (1996). Empathy: A Social Psyhoclogical Approach.

[B25] Nakane C (1970). Japanese Society.

[B26] Hoshino K (1995). Autonomous decision making and Japanese tradition. Camb Q Healthc Ethics.

[B27] Voltz R, Akabayashi A, Reese C, Ohi G, Sass HM (1998). End-of-life decisions and advance directives in palliative care: a cross-cultural survey of patients and health-care professionals. J Pain Symptom Manage.

